# Linguistic feature analysis for protein interaction extraction

**DOI:** 10.1186/1471-2105-10-374

**Published:** 2009-11-12

**Authors:** Timur Fayruzov, Martine De Cock, Chris Cornelis, Veronique Hoste

**Affiliations:** 1 Ghent University, Department of Applied Mathematics and Computer Science, Krijgslaan 281 (S9), 9000 Gent, Belgium; 2 University College Ghent, School of Translation Studies, Groot-Brittanniëlaan 45, 9000 Gent, Belgium; 3 University of Washington, Institute of Technology, 1900 Commerce Street, Tacoma, WA-98402, USA

## Abstract

**Background:**

The rapid growth of the amount of publicly available reports on biomedical experimental results has recently caused a boost of text mining approaches for protein interaction extraction. Most approaches rely implicitly or explicitly on linguistic, i.e., lexical and syntactic, data extracted from text. However, only few attempts have been made to evaluate the contribution of the different feature types. In this work, we contribute to this evaluation by studying the relative importance of deep syntactic features, i.e., grammatical relations, shallow syntactic features (part-of-speech information) and lexical features. For this purpose, we use a recently proposed approach that uses support vector machines with structured kernels.

**Results:**

Our results reveal that the contribution of the different feature types varies for the different data sets on which the experiments were conducted. The smaller the training corpus compared to the test data, the more important the role of grammatical relations becomes. Moreover, deep syntactic information based classifiers prove to be more robust on heterogeneous texts where no or only limited common vocabulary is shared.

**Conclusion:**

Our findings suggest that grammatical relations play an important role in the interaction extraction task. Moreover, the net advantage of adding lexical and shallow syntactic features is small related to the number of added features. This implies that efficient classifiers can be built by using only a small fraction of the features that are typically being used in recent approaches.

## Background

Nowadays, an overwhelming amount of experimental studies on gene and protein interactions are being conducted. The results of these experiments are most often described as scientific reports or articles and published in public knowledge repositories, such as Medline http://www.ncbi.nlm.nih.gov/. This literature database grows at a rate of 2000 publications per week, which makes it impossible for a human to track every new experiment performed in the field.

Therefore, the need for automated information extraction methods in biomedicine becomes critical, and a lot of efforts are invested in creating such methods. Recently proposed approaches for interaction extraction are based not only on explicit textual information that is contained in publications, but also on a comprehensive language analysis that includes part-of-speech (POS tags) and deep syntactic structure detection. To achieve state-of-the-art performance, researchers employ lexical information (words) along with shallow syntactic information (POS) and/or deep syntactic features (grammatical structures) (see for example [1-10]).

As a consequence, extraction methods tend to become more complex, use more features and require more and more memory and computational efforts. However, little attention has been devoted to studying the individual impact of different feature types. We believe that this question is of great importance, because (1) when two types of features have a substitute rather than a complementary effect, one of them can be dropped to obtain a computationally more efficient method, and (2) dropping one type of features might make the mining algorithm more robust. The latter reason is especially relevant for lexical features since lexicons tend to be subdomain-specific. This problem can be alleviated by combining different biological phenomena in one corpus; however in practice corpora are often built for a particular organism or a particular set of proteins. Despite this fact, it is common practice in the field to train and evaluate systems on the same data set with an n-fold cross-validation technique, thus partially avoiding this lexicon-dissimilarity problem which is inherent to real-life problems.

In this work, we study the impact of different feature types on the performance of a relation extraction system that uses a support vector machine (SVM) classifier with kernels as its core, since at present this is the most popular choice in the relation extraction field. In particular, we use the approach suggested by Kim et al. [6], which relies on lexical, shallow and deep syntactic features represented as parts of a dependency tree, and consequently apply Occam's razor principle by cutting off the former two to get rid of all lexical and shallow syntactic information. In other words, we would like to exploit different aspects of the dependency tree and compare the net advantage that is obtained by these feature types.

To the best of our knowledge, besides us, only [7-9] have looked into the impact of syntactic in addition to lexical features for the protein interaction extraction task (all in the context of SVMs). Shallow syntactic features such as POS added to a lexical feature set are reported not to increase the performance of the classifier in [9], while the deep+shallow syntactic- and lexical-feature based classifier in [7] showed a poor performance when the set of lexical features is limited. Neither of these has however studied how much performance can be obtained by using *only *deep syntactic features. The closest to our work is [8] which compares the performance of an interaction extraction system using only lexical features versus using syntactic (both shallow and deep) features. We highlight the difference with our work at the end of Section 'Related Work'.

The contribution of this article is twofold. First, we perform an extensive evaluation of a recently published SVM-based approach [6], which was evaluated only on the LLL data set before, on 5 data sets (AIMed [11], BioInfer [12], HPRD50 [3], LLL [13] and IEPA [14]) using cross-validation as well as 10 cross-data set experiments. Secondly, we compare this approach with stripped down versions which take into account different feature subsets, and we demonstrate that omitting lexical and part of the syntactic features does not significantly change the performance of the relation extraction task.

In the remainder of this paper, we first formalize the protein interaction extraction problem as a classification task (Section 'Problem Statement') in which sentences containing protein pairs are represented by dependency trees (Section 'Interaction Representation'). In Section 'Building a classifier', we present the various classifiers that we use in this paper, all of them modifications of [6], and in Section 'Related Work' we clarify the relationship with related methodologies. We continue with a description of our experimental setup and present the results on the different data sets in Section 'Results and Discussion'. Our final conclusions are presented in Section 'Conclusion'.

## Methods

### Problem statement

Whereas the general interaction extraction task is concerned with finding all interactions among proteins in a given text, several assumptions are usually made to simplify it. The first assumption is that the extraction task is restricted to binary interactions, i.e., exactly two proteins are involved in the interaction. Secondly, the interaction is assumed to be fully expressed in one sentence, i.e., interactions which are described across several sentences are not considered. Finally, the interaction extraction task is evaluated separately from the protein name recognition task. Named entity recognition (NER) is another area of text mining, which is usually performed and evaluated separately, thus it is generally assumed that interaction extraction is performed on a text with annotated protein names ([2,3,5,15]).

Let us consider the following sentence containing 4 protein names.

Example 1: "In the **shaA**_**1 **_mutant, **sigma(H)**_**2**_-dependent expression of **spo0A**_**3 **_and **spoVG**_**4 **_at an early stage of sporulation was sensitive to external NaCl."

This sentence contains 6 protein pairs, namely *shaA-sigma(H)*, *shaA-spo0A*, *shaA-spoVG*, *sigma(H)-spo0A*, *sigma(H)-spoVG*, and *spo0A-spoVG*. A protein pair is a positive instance if the original sentence expresses an interaction between members of this pair, and a negative instance if they just co-occur in the sentence. In the example above, there are two positive instances, namely *sigma(H)-spo0A *and *sigma(H)-spoVG *while the other 4 instances are negative. As such, the task of protein interaction extraction can be treated as a classification problem, to be solved by learning a suitable decision function that can separate the positive from the negative instances.

In particular, we need to choose a formal protein pair representation and a machine learning algorithm. The protein pair representation should include information from the sentence that can be used to distinguish between positive and negative instances. Different approaches use different types of information (features), depending on the machine learning methods used, the available tools and the researcher's strategy. Although feature selection for interaction extraction has received little attention [16], several researchers [8,17] report that applying feature selection techniques significantly speeds up the processing and in some cases increases the performance of the classifier. The difference between the feature selection problem and the current approach is explained in detail in Section 'Related Work'.

### Interaction representation

**Figure 1:**
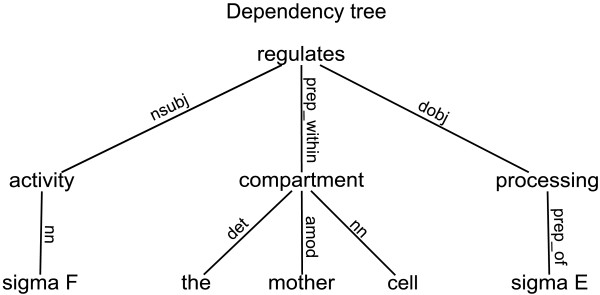
**Dependency tree for the sentence from Example 1**. Figure 1 represents the dependency tree for the following sentence: "**Sigma F**_**1 **_activity regulates the processing of **sigma E**_**2 **_within the mother cell compartment."

A dependency tree represents the syntactic structure of a sentence. The nodes of the tree are the words of the sentence, and the edges represent the dependencies between words. In a typed dependency tree, edges are labeled with syntactic roles. The dependency tree for the following sentence is depicted in Figure 1.

Example 2: "**Sigma F**_**1 **_activity regulates the processing of **sigma E**_**2 **_within the mother cell compartment."

**Figure 2:**
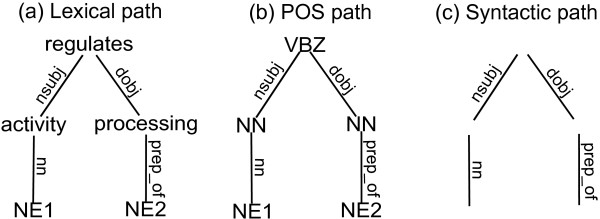
**Shortest path dependency trees for the protein pair in the sentence from Example 2**. Figure 2a represents the shortest path between the two proteins in the dependency tree for the sentence: "**Sigma F**_**1 **_activity regulates the processing of **sigma E**_**2 **_within the mother cell compartment." Figures 2b and 2c represent respectively the corresponding POS shortest path and the syntactic path. Note that 2a contains lexical and deep syntactic information, while 2b contains shallow and deep syntactic information, and 2c contains only deep syntactic information.

**Figure 3:**
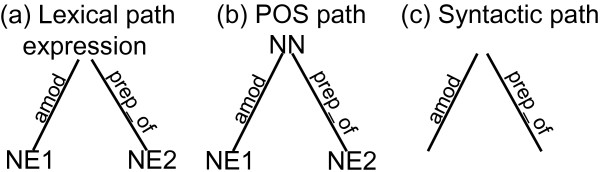
**Shortest path dependency trees for one of the protein pairs in the sentence from Example 1**. Figure 3 depicts the shortest path dependency trees for the protein pair *sigma(H)-spo0A *built from the dependency tree of the sentence: "In the **shaA**_**1 **_mutant, **sigma(H)**_**2**_-dependent expression of **spo0A**_**3 **_and **spoVG**_**4 **_at an early stage of sporulation was sensitive to external NaCl."

The most relevant part of the dependency tree to collect information about the relation between the two proteins is the subtree corresponding to the shortest path between these proteins, which is shown in Figure 2a. Both protein names are replaced with dummy strings *NE*_1 _and *NE*_2 _in order to generalize the interaction pattern. Moreover, we introduce a POS dependency tree, where nodes represent part-of-speech information instead of the corresponding words. The shortest path between the two proteins in the POS dependency tree for Example 2 is represented in 2b. Note that a dependency tree contains lexical as well as deep syntactic information, while a POS dependency tree contains shallow and deep syntactic information. We can obtain a syntactic shortest path by only retaining the syntactic roles in either the shortest path or the POS shortest path, as shown in Figure 2c. Figure 3 depicts similar information for the sentence from Example 1.

In the remainder we keep referring to these paths as (shortest path) dependency trees. Such a dependency tree can be either a lexical dependency tree (like Figure 2a or 3a), a POS dependency tree (like Figure 2b or 3b) or a syntactic dependency tree (like Figure 2c or 3c). We will use *t *= (*N*, *E*, *L*) to denote a dependency tree *t *consisting of a set of nodes *N*, a set of edges *E*, and a function *L *that maps nodes and edges to their labels. If there is an edge from node *n*_1 _to node *n*_2_, we denote this edge by *e*(*n*_1_, *n*_2_).

### Building a classifier

Our feature impact study makes use of a support vector machine (SVM) approach. SVM's are a classification method that views input data as vectors in a high-dimensional space and attempts to induce a maximum margin hyperplane between training data points that belong to different classes. The crucial point is that the hyperplane can be represented as a linear combination of a subset of training instances (support vectors) [18]. Moreover, the selection of the appropriate support vectors can be done by using only the inner product between training examples. Hence, if the inner product can be computed efficiently, SVM's can induce a classifier even in a very rich feature space. In order to deal with various kinds of input data, different strategies to compute inner products (referred to as kernels) have been proposed (see e.g. [19]).

In order to build a syntactic kernel, the dependency tree space has to be kernelized. Each data point of our data set is a dependency tree corresponding to a part of the sentence in which the protein pair occurs. Such a tree can be represented as a vector in the *m*-dimensional space made up by all subtrees in the data set [20]. More in particular, assuming that all unique subtrees in the data set are enumerated from 1 to *m*, the function *h*_*s*_(*t*), *s *∈ {1,...,*m*}, is defined as the number of occurrences of subtree *s *in tree *t*. Then, each tree *t *can be represented by a vector *ϕ *(*t*) = {*h*_1_(*t*), *h*_2_(*t*),...,*h*_*m*_(*t*)}. A kernel function measuring the similarity between trees *t*_1 _and *t*_2 _based on whether they contain the same subtrees, is defined as the inner product(1)

The underlying vector representation is very rich since the number of subtrees of a tree grows exponentially with the tree size, which makes the computation of the inner product intractable. However, the right hand side of (1) can be interpreted as the number of common subtrees of *t*_1 _and *t*_2_, and can be computed efficiently following a procedure proposed in [20].

Kim et al. [6] follow this procedure in developing an SVM classifier based on a kernel *K*_*FULL *_that is a combination of a kernel *K*_*LEX *_comparing lexical dependency trees, with a kernel *K*_*POS *_comparing POS dependency trees. Note that, because of their construction, *K*_*LEX *_relies on lexical and deep syntactic information, while *K*_*POS *_is based on shallow and deep syntactic features. We propose a way in which the kernel *K*_*FULL *_can be stripped down to a kernel *K*_*S*_, that uses only deep syntactic features. We compare the performance of all these kernels in Section 'Results and Discussion'. For ease of explanation, in this section we follow a bottom-up approach by first defining *K*_*S*_, and then extending it to the full system from [6].

The trees from Figures 2a and 3a have no subtrees in common, while when we switch to a shallow or pure syntactic representation in Figures 2b,c and 3b,c, we have one common fragment, namely the subtree consisting only of the edge *prep_of *and its adjacent nodes. In general, we use a recursive formula to compute the number of common subtrees between dependency trees *t*_1 _and *t*_2_. This formula relies on the notion of *common child pairs *of node *n*_1 _in *t*_1 _and node *n*_2 _in *t*_2_, i.e. the set of pairs of nodes that have parents *n*_1 _and *n*_2 _respectively, and that are connected to these parents by the same type of edge. When traversing down the trees in search of common subtrees, these are the nodes at which we want to continue our exploration.

*Definition 1*. Let *t*_1 _= (*N*_1_, *E*_1_, *L*_1_) and *t*_2 _= (*N*_2_, *E*_2_, *L*_2_) be dependency trees. For *n*_1 _∈ *N*_1 _and *n*_2 _∈ *N*_2_, the set of common child pairs is defined as *Com*(*n*_1_, *n*_2_) = {(*x*, *y*)| (*x*, *y*) ∈ *N*_1 _× *N*_2_, *e*(*n*_1_, *x*) ∈ *E*_1_, *e*(*n*_2_, *y*) ∈ *E*_2_, *L*_1_(*e*(*n*_1_, *x*)) = *L*_2_(*e*(*n*_2_, *y*))}.

*Definition 2*. Let *t*_1 _= (*N*_1_, *E*_1_, *L*_1_) and *t*_2 _= (*N*_2_, *E*_2_, *L*_2_) be dependency trees. For *n*_1 _∈ *N*_1 _and *n*_2 _∈ *N*_2_, the number of common subtrees rooted at *n*_1 _and *n*_2 _is defined as

The recursive formula reflects the fact that a new common subtree rooted at *n*_1 _and *n*_2 _can be found either by picking 1 of the *Cm*(*x*, *y*) subtrees or by adding the *x*/*y *nodes, or just by staying as is (therefore +2). 1 is subtracted from the whole result to exclude the combination with the tree consisting of the *n*_1_/*n*_2 _node only.

*Example 3*. Let *t*_1 _and *t*_2 _be the dependency trees from Figure 2a and 3a respectively. *Com*(*n*_1_, *n*_2_) is the empty set for all node pairs with exception of *Com*(*processing, expression*) = {(*NE*2, *NE*2)}. Hence *Cm*(*processing, expression*) = (*Cm*(*NE*2, *NE*2) + 2) -1 = 1 while *Cm*(*n*_1_, *n*_2_) = 0 for all other node pairs. This means that there is only one common subtree between *t*_1 _and *t*_2_, rooted at the *processing, expression *nodes and ending at *NE*2.

Note that the calculation above of the number of common subtrees disregards node labels, i.e., it treats dependency trees as they are shown in Figure 2c and Figure 3c. Using Definition 2 we are now able to define a kernel *K*_*S *_that looks only at deep syntactic information. It computes the similarity between syntactic dependency trees as the number of grammatical structures that they have in common.

*Definition 3*. The kernel function *K*_*S *_is defined as(2)

for syntactic dependency trees *t*_1 _and *t*_2_.

*Example 4*. Let *t*_1 _and *t*_2 _be the syntactic dependency trees from Figure 2c and Figure 3c respectively. Since |*N*_1_| = 5 and |*N*_2_| = 3, the summation in the right hand side of (2) consists of 15 terms. In Example 3 we already established that all of these terms are 0 with the exception of one term that equals 1. Hence *K*_*S*_(*t*_1_, *t*_2_) = 1.

To arrive at kernels that take into account additional lexical and/or shallow syntactic information, we need an extended version of Definition 1 that also looks at the labels of nodes.

*Definition 4*. Let *t*_1 _= (*N*_1_, *E*_1_, *L*_1_) and *t*_2 _= (*N*_2_, *E*_2_, *L*_2_) be dependency trees. For *n*_1 _∈ *N*_1 _and *n*_2 _∈ *N*_2_, the set of common child pairs, taking into account the labels of the nodes, is defined as *Com*^*lab*^(*n*_1_, *n*_2_) = {(*x*, *y*)|(*x*, *y*) ∈ *Com*(*n*_1_, *n*_2_), *L*_1_(*n*_1_) = *L*_2_(*n*_2_), *L*_1_(*x*) = *L*_2_(*y*)}.

The superscript "*lab*" refers to the fact that the labels of the nodes are taken into account. The appearance of *Com*(*n*_1_, *n*_2_) in the definition of *Com*^*lab*^(*n*_1_, *n*_2_) illustrates that the latter builds on the former. Furthermore, it holds that

indicating that using syntactic trees leads to a more general approach (more nodes are explored when traversing down the trees in search for common subtrees).

The number *Cm*^*lab*^(*n*_1_, *n*_2_) of common subtrees rooted at *n*_1 _and *n*_2_, can now be defined in a recursive manner entirely analogous to Definition 2, however relying on *Com*^*lab*^(*n*_1_, *n*_2_) instead of on *Com*(*n*_1_, *n*_2_). Since they have different labels at the nodes, the value of *Cm*^*lab*^(*n*_1_, *n*_2_) might be different depending on whether a lexical dependency tree or a POS dependency tree is used. In both cases, it holds however that(3)

*Example 5*. Let *t*_1 _and *t*_2 _be the lexical dependency trees from Figure 2a and Figure 3a respectively. For all node pairs it holds that *Com*^*lab*^(*n*_1_, *n*_2_) = ∅, and *Cm*^*lab*^(*n*_1_, *n*_2_) = 0.

*Example 6*. Let *t*_1 _and *t*_2 _be the POS dependency trees from Figure 2b and Figure 3b respectively. It holds that *Com*^*lab*^(*NN, NN*) = {(*NE*2, *NE*2)} and *Cm*^*lab*^(*NN, NN*) = 1, while for all other node pairs *Com*^*lab*^(*n*_1_, *n*_2_) = ∅ and *Cm*^*lab*^(*n*_1_, *n*_2_) = 0.

The potentially different behavior of *Cm*^*lab*^(*n*_1_, *n*_2_) on lexical dependency trees and POS dependency trees gives rise to the definitions of the kernel functions *K*_*LEX *_and *K*_*POS *_respectively. Both of them still consider the tree structure when computing the similarity between trees, i.e. they both rely on deep syntactic information. In addition, *K*_*LEX *_takes the actual words of the sentence into account (lexical information) while *K*_*POS *_considers POS (shallow syntactic information).

*Definition 5*. [20] The kernel function *K*_*LEX *_is defined as(4)

for lexical dependency trees *t*_1 _and *t*_2_. In our case function *L *maps words in the tree nodes to corresponding lemmas eliminating the differences arising from different word forms.

*Definition 6*. [6] The kernel function *K*_*POS *_is defined as

for POS dependency trees *t*_1 _and *t*_2_.

Finally, Kim et al. [6] combine *K*_*LEX *_and *K*_*POS *_into a kernel *K*_*FULL *_that takes into account lexical, shallow and deep syntactic information.

*Definition 7*. [6] The kernel *K*_*FULL *_is defined as(5)

for dependency trees *t*_1 _and *t*_2 _and their corresponding POS dependency trees  and .

Notice that *K*_*LEX *_is a refinement of *K*_*S *_in the sense that all the information used by *K*_*S *_is also used in the same way by *K*_*LEX*_. As a consequence, *K*_*FULL *_is also a refinement of *K*_*S*_, enriching the deep syntactic information of *K*_*S *_by lexical information (through *K*_*LEX*_) as well as shallow syntactic information (through *K*_*POS*_).

*Example 7*. Let *t*_1 _and *t*_2 _be the lexical dependency trees from Figure 2a and Figure 3a respectively, and  and  their corresponding POS dependency trees from Figure 2b and Figure 3b respectively. One can verify that

and

hence *K*_*FULL*_(*t*_1_, *t*_2_) = 1. Notice that although the trees do not show any resemblance on the lexical level, their similarity at the more general syntactic level is picked up by *K*_*POS*_. In Example 4 we found that their syntactic similarity is also already reflected by *K*_*S*_.

A short summary of the kernels described above is provided in Table 1.

**Table 1:** Kernels

Notation	Formula	Used information
*K*_*S*_(*t*_1_, *t*_2_)		Deep syntactic
*K*_*LEX*_(*t*_1_, *t*_2_)		Lexical + deep syntactic
*K*_*POS*_(*t*_1_, *t*_2_)		Shallow + deep syntactic
*K*_*FULL*_(*t*_1_, *t*_2_)	*K*_*LEX *_+ *K*_*FULL*_	Lexical + Shallow + deep syntactic

### Related work

In Table 2, an overview of recent approaches to interaction extraction is presented along with the characteristics that are relevant in the context of our work. Below we describe these approaches in more detail.

**Table 2:** General approaches for protein interaction extraction

Method	Information	Algorithm	Data sets
[2]	lexical	SVM	AIMed

[4]	lexical	SVM	AIMed
	shallow		LLL

[9]	lexical	Maximum entropy	IEPA
	shallow		
	deep		

[10]	lexical	SVM	AIMed
	shallow		
	deep		

[5]	lexical	BayesNet	AIMed
	deep	NaiveBayes	
		K-nearest neighbour	
		Ensembles	

[3]	lexical	Hand-built rules	HPRD50
	shallow		LLL
	deep		

[15]	shallow	C4.5	AIMed
	deep	BayesNet	LLL

[7]	lexical	SVM	AIMed
	deep		

[1]	lexical	Sparse RLS	AIMed
	shallow		BioInfer
	deep		HPRD50
			IEPA
			LLL

[6]	lexical	SVM	LLL
	shallow		
	deep		

[8]	lexical	SVM	AIMed
	shallow		HPRD50
	deep		IEPA
			LLL

Many approaches exploit the idea of using explicit feature vectors to represent a possible interaction. In particular, approaches based on various combinations of lexical features are very popular in the relation extraction community.

Bunescu et al. [2] propose to use the sentence context, obtained by splitting a sentence into three parts, i.e. before the first protein, between the two proteins, and after the second protein, and they combine them in predefined ways to obtain 3 types of patterns. Using this information, the authors propose a kernel that naturally emerges from the subsequence kernel described in [21] and obtain good results on the AIMed corpus. Giuliano et al. [4] start from the same pattern types, but treat them as bags-of-words, and define a global context kernel. Moreover, they define a local context kernel by taking a window of predefined size around the candidate proteins and adding more shallow linguistic information, such as the lemma of the word and some orthographic features. The resulting kernel function in this case is a linear combination of the global context kernel and the local context kernel. Their method obtains state-of-the-art results on the AIMed and LLL data sets.

Some researchers focus on sentence structure, i.e., on the parse and dependency tree, to construct a feature vector. Xiao et al. [9] study the impact of features, starting with simple words up to parse and dependency trees on the IEPA corpus, and they obtain a remarkable 90.9% F-score using a maximum entropy model with lexical and shallow syntactic features. Yakushiji et al. [10] suggest that full parsing information could be very useful in the biology domain because the distance between entities in a sentence can be much longer than in general-purpose domains. Therefore, they propose a method that builds complex predicate-argument structures (PAS), and apply an SVM with an RBF kernel to these patterns to obtain a classifier model. They evaluate this model on the AIMed data set and obtain a 57.3% F-score. In [5], the authors also focus on sentence structure and use dependency trees to extract the local contexts of the protein names, the root verbs of the sentence, and the parent of the protein nodes in the dependency tree. Classification is further done by BayesNet and ensemble classifiers. Another approach is proposed in [3], where a manually constructed set of rules uses information from the dependency trees and a predefined vocabulary to classify possible interaction instances. This approach is evaluated on the HPRD50 and LLL data sets, as well as on a large-scale data set consisting of 1 million MEDLINE abstracts. The extracted set of interactions contained 40% of the HPRD interaction database.

In our own previous work [15], we proposed to abstract from lexical features and use only syntactic information to obtain a more general classifier that would be suitable for different data sets without retraining. We used features extracted from dependency and parse trees to build decision trees and BayesNet classifiers, and obtained promising results using AIMed as test data and LLL as training data. Another group of approaches does not rely on an explicit feature vector but rather makes use of structured data as input information for the classifier. This means that structured features, such as dependency trees, can be used as an input to the classifier without any additional transformations, thus reducing the risk of losing useful information. One particular way to use structured features that we adhere to in the current paper, is to exploit structured kernels.

The approaches [1,6,7] are closest to our work as they also use structured kernels. Structured or convolution kernels were introduced in [22] by Haussler who proposed how to compute a kernel for structured objects. This work gave rise to many tree kernel methods in the text mining domain. Although this idea is quite popular in general text mining, it has not been widely explored in the interaction extraction literature.

Saetre et al. [7] apply a structured kernel to the protein-protein interaction domain. In this approach, a mix of at and structured features is used to calculate the similarity of two protein pairs. The flat part of the feature vector contains lexical features, while the structured part is a shortest path dependency tree, referred to as a partial tree. This definition was introduced by Moschitti [23] who studied different tree partitioning strategies and their impact on tree kernels for dependency and parse trees. Using these fatures, Saetre et al. obtain promising results on the AIMed data set, especially in combination with a rich lexical feature set.

In another very recent approach, described in [1], the authors propose to use the whole dependency tree to build a classifier. They use a graph kernel that takes into account all paths in dependency trees, and exploit it with an RLS (regularized least squares) machine learning method. The experimental evaluation is performed on 5 data sets: AIMed, BioInfer, HPRD50, IEPA and LLL, and for all of them, the method shows remarkably good results.

Collins and Duffy [20] developed a tree kernel that counts the number of common subtrees, but used it for parsing and not for interaction extraction. Kim et al. [6] apply this kernel to a dependency tree and to a modified dependency tree with POS instead of words, and propose a combined kernel, which is a sum of these two. This approach obtains state-of-the-art performance on the LLL data set. In the same paper they describe a flat feature vector-based approach that also utilizes dependency trees to extract graph walks as features. In [8], the authors study the relative feature importance for the latter approach by using the gain ratio feature selection technique. Moreover, they study the impact of different feature types as well by comparing the performance of methods that use syntactic features versus methods that use lexical features. Our approach is also based on Kim's work, however it is different from [8] in several aspects. First of all, our approach is different from the feature selection task, because we focus on the *type *of the information (lexical, POS, grammatical relations) rather than on separate features. In other words, we do not use feature selection techniques to discriminate useful individual features, but fit an existing relation extraction method to consider only a subset of features of a certain type, and study the impact of this feature class. Secondly, when we study the impact of different feature types we do not rely on a flat vector, but on a structured representation. Moreover, we use an additional data set and define more extensive experimental setups in order to perform a complete study of different use cases.

## Results

### Data sets

To the best of our knowledge the only publicly available data sets that contain protein interaction annotations are: AIMed [11], BioInfer [12], HPRD50 [3], LLL [13] and IEPA [14]. These data sets have been frequently used in recent work of for example [1,8,24]; therefore we use them in our current work. Table 3 gives an overview of the number of positive and negative instances in the different data sets.

**Table 3:** Corpora statistics

Data set	# of interaction instances	
	positive	negative
AIMed	1057	4790
BioInfer	1381	8964
HPRD50	163	270
LLL	164	166
IEPA	411	482

The AImed data set consists of 225 abstracts extracted from the Database of Interaction Proteins (DIP), 200 of which contain annotated human gene and protein interactions. Another 25 abstracts contain protein names but do not describe any interactions. We have used only the former set of abstracts for our evaluation purposes.

The BioInfer data set is the largest data set among these 5; it contains 1100 sentences describing protein-protein interactions. Beside the interaction annotations, BioInfer contains additional information about biological interaction type, protein roles in interaction, syntactic dependencies between words, etc. Moreover, there is a knowledge base behind the corpus, which allows to analyse it in more detail (see [12]). HPRD50 contains sentences that were extracted from a subset of 50 abstracts, referenced by the Human Protein Reference Database (HPRD) and annotated with protein names and interactions between them. The LLL data set consists of 76 sentences describing interactions concerning *Bacillus subtilis *transcription. Protein roles for interactions are annotated along with the interactions themselves. Additionally, the data set contains annotations for lemmas and syntactic dependencies between the words in the sentences. Finally, The IEPA data set was built by querying Medline with 10 diverse queries, reflecting 10 different biological topics. 303 abstracts were retrieved, and a data set was constructed with sentences extracted from these abstracts. The data set annotation includes an interacting verb along with the protein names and interactions.

The BioInfer and LLL data sets provide syntactic dependencies for every sentence in their own formats, while the other data sets do not provide this information. We discarded this information to unify the setup and to make the experiment more realistic. Besides being non-standard, some of the syntactic information in BioInfer and LLL was obtained manually which violates the requirements of automatic processing. To obtain POS and dependency trees for all data sets we used the Stanford parser [25] trained on general purpose corpora. We choose this parser because of its peculiar annotation scheme that stresses the semantic connections between words rather than operating on the purely syntactic level. For example, prepositions are collapsed as can be seen in Figure 2a, where the noun *processing *is being connected directly to a protein name. As we use a dependency tree representation to obtain all three types of features, we do not use an external POS tagger to obtain POS tags separately; instead they are assigned as part of the dependency tree building process inside the Stanford parser. As the Stanford parser does not provide lemmatized versions of words, we used the Porter Stemmer algorithm [26] to compute *K*_*LEX *_and *K*_*FULL*_. All data sets use different annotation schemes that emphasize different interaction properties. For example, in AIMed homodimeric proteins are being annotated, i.e. proteins that interact with themselves, while the current mining approach is not able to detect such cases. Moreover, in BioInfer some proteins have gaps in annotations, i.e. there is a gap between two parts of one protein name. We handle these cases separately, but they can potentially decrease the performance of the classifier as well. The quality of the annotation itself (measured as e.g. inter-annotator agreement) may affect the quality of classifier. If an annotator misses an interaction between two proteins, the data point related to this protein pair would be treated as a negative instance, although containing an interaction pattern, which is harmful to the overall performance. To unify an experimental setup, we need to cast all corpora to a common ground format. Pyysalo et al. [27] designed custom software that converts all 5 data sets to a single XML format that contains only minimal protein and interaction annotations, which is sufficient for our evaluation purposes. However, not all annotation differences can be eliminated in this way. Table 3 shows that different data sets have very different positive/negative ratios. This can be partially explained by different annotation strategies, e.g. for LLL only proteins that are involved in interactions are annotated, while for other data sets all protein names are annotated. Since we consider every possible protein pair within a sentence to be an instance, this leads to an exponential growth of the total number of instances, while in fact the number of positive instances remains the same. Taking into account this information, we should choose our evaluation metrics carefully in order to provide a fair comparison of the performance on all data sets.

### Performance metrics

In this work, we use two evaluation metrics, namely recall-precision and ROC (reciever operating characteristic) curves, to provide a more comprehensive analysis of the achieved results. Let us first recall their definitions. Let *TP *denote the number of true positives, i.e., the number of positive instances that are classified as such, let *FP *denote the number of false positives, i.e., the number of negative instances that are incorrectly classified as positive, and analogously, let *TN *and *FN *stand for the number of true negatives and false negatives respectively. The following metrics can then be defined:

Recall stands for the fraction of correctly classified instances (TP) among all positive instances (TP+FN) in a data set, while precision denotes the fraction of correctly classified instances (TP) among all instances that are classified as positive (TP+FP). Recall is sometimes called true positive rate, while false positive rate counts how many of the negative instances were wrongly classified as positive. A combined measure, that takes into account both recall and precision is called F-score and defined as:

Often, a classifier's output can be ordered, i.e. the classifier also provides a degree of confidence for each prediction it makes. In this case, we can trade precision for a higher recall by lowering the confidence threshold to capture more positive instances. In this way we can build a recall-precision curve that shows the relationship between the quality of extracted relations (precision) and the amount of extracted relations (recall). The closer to the top-right corner a curve is, the less precision is lost with recall growth and the better the performance of the classifier is.

Precision, recall and F-score are de-facto standards for the interaction extraction evaluation. However, these metrics are very sensitive to data set skewedness, i.e., the large difference between the number of positive and negative instances. As was shown in Table 3, this difference varies greatly for different corpora. On the other hand, ROC curves are being used in the machine learning community to evaluate classifier performance and they do not depend on data set skewedness.

The false positive rate together with the true positive rate correspond to a point in ROC space. By varying the trade-off between these two metrics we obtain a curve in ROC space. The AUC-score is the area under this ROC-curve. It can be interpreted as the probability that the classifier will rank a randomly chosen positive instance higher than a randomly chosen negative instance.

However, this metric should be used carefully for the same reason, i.e. it is suitable to evaluate the relative quality of a classifier (percentage of extracted positive instances), but it gives no information about precision, and thus makes the evaluation of a classifier difficult.

For example, if we increase the number of negatives 10 times, then the number of FP on average increases 10 times as well. This will lead to a significant drop in precision and consequently in F-score, but it does not influence the false positive rate.

Based on this observation, we can outline an application area for both evaluation metrics. The ROC curve and the corresponding AUC value should be used to compare the performance of a classifier on different corpora, since they show the relative number of extracted positive instances. Recall-precision and F-score can be used to compare the quality of several classifiers on the same data set, since they indicate how 'clean' the classification is without regarding the proportion of negative instances.

### Experimental setup

For the experiments, we used the LIBSVM library [28] to build the 4 SVM classifiers that use the *K*_*FULL *_kernel, *K*_*POS *_kernel, *K*_*S *_kernel and *K*_*LEX *_kernel. Furthermore, we organized 3 experimental setups. The first setup uses 10-fold cross-validation (CV), where each data set is split into 10 parts, of which 9 are used for training and one for testing. Despite the fact that this is the most common way of evaluation, it should be used carefully. Since we work on instance level, it can be the case that two nearly identical instances from the same sentence fall into a train and a test fold at the same time. This 'leak' can cause a performance boost as it was shown in [7,8].

In the second setup (4-1) we join 4 data sets to form a training set, and use the remaining one as a test set. Compared to CV, this alternative experimental setup is closer to a real world situation where information for processing is obtained from different sources and the lexicon is not as uniform as in one precompiled data set.

In the last setup (1-4) we use 1 data set as training set and the remaining 4 as test sets, thus making another step to the real world. Typically, biologists have a very limited amount of annotated data compared to the size of available unlabeled information. We try to model this situation here by making the training set much smaller than the test set.

For each experimental setup we run all classifiers with all data set combinations. An analysis of the results obtained is provided in the following section.

## Discussion

**Figure 4:**
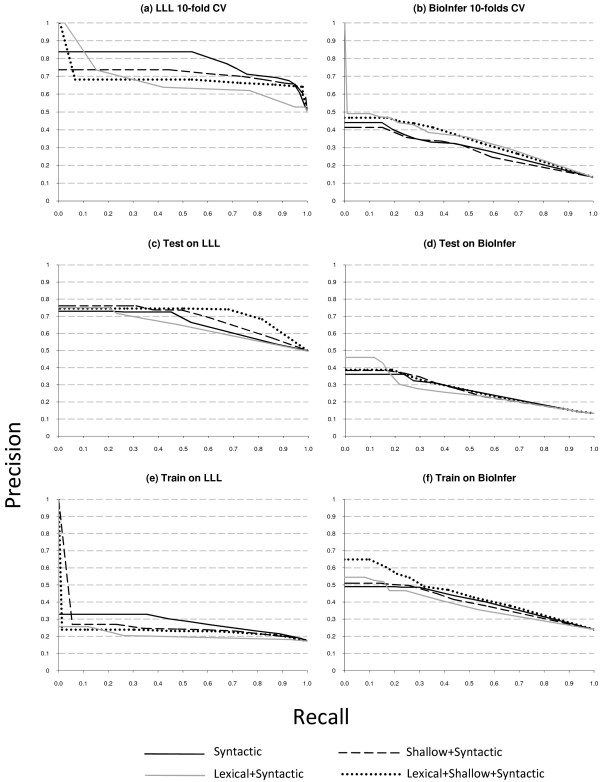
**Recall-precision curves for all experimental setups for LLL and BioInfer**. The left charts represent LLL-related curves; the right charts BioInfer-related curves.

**Figure 5:**
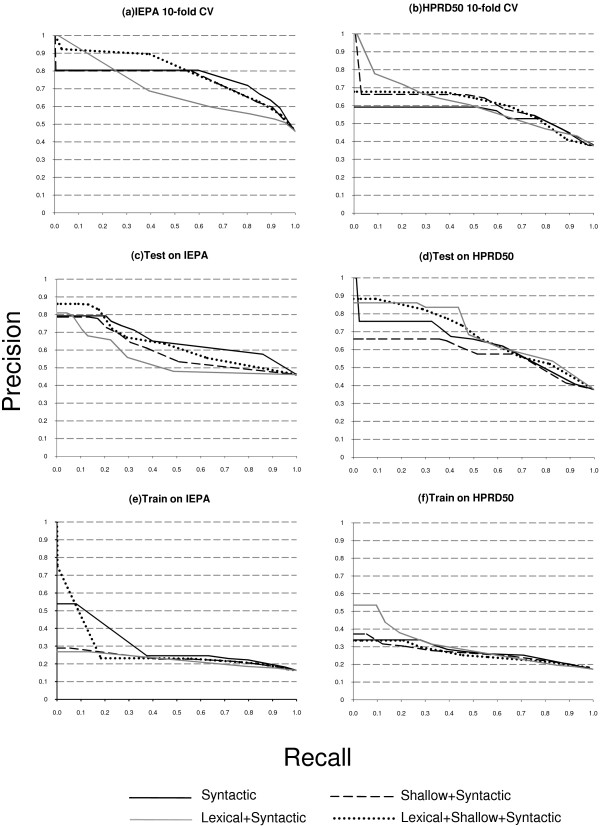
**Recall-precision curves for all experimental setups for IEPA and HPRD50**. The left charts represent IEPA-related curves; the right charts HPRD50-related curves.

**Figure 6:**
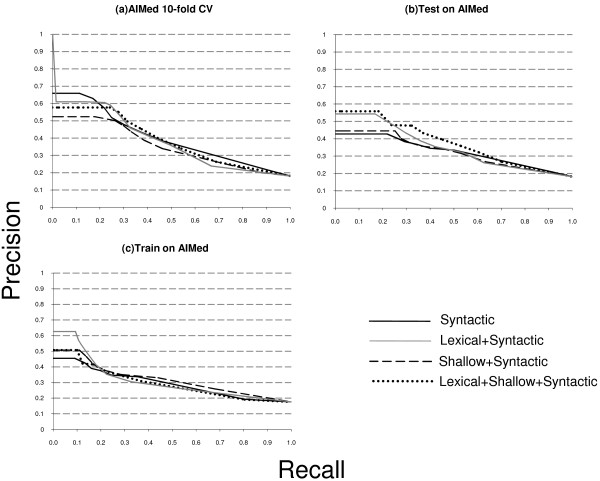
**Recall-precision curves for all experimental setups for AIMed**. The charts represent AIMed-related curves.

**Figure 7:**
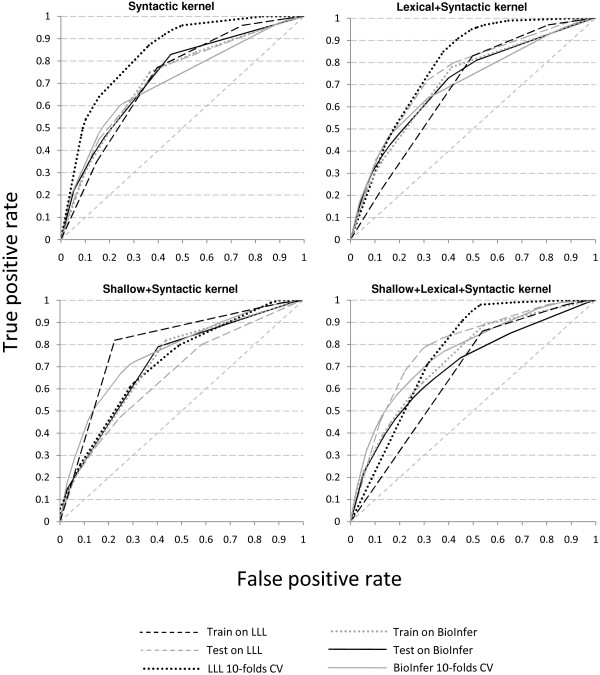
**ROC curves for different classifiers**. Figure 7 depicts ROC curves for different kernels. From left top to right bottom: lexical kernel, Lexical+Shallow kernel, shallow kernel, syntactic kernel.

Table 4 and Figures 4, 5, 6, 7 give an overview of the evaluation results for all experimental setups. In line with our evaluation metric review, we use recall-precision and ROC curves to analyze the obtained experimental results. On the basis of these results, we can make the interesting observation that the different kernels are roughly comparable, while the amount of information they use is very different. In the analysis below we will omit the +syntactic postfix when talking about the lexical+syntactic (*K*_*LEX*_), shallow+syntactic (*K*_*POS*_) and lexical+shallow+syntactic (*K*_*FULL*_) kernels.

**Table 4:** Results

Data set	**Exp**.	Synt. kernel		Shallow+Synt. kernel		Lex.+Synt. kernel		Shallow+Lex.+Synt. kernel	
		F-score	AUC	F-score	AUC	F-score	AUC	F-score	AUC
AIMed	CV	0.33	0.69	0.37	0.66	0.37	0.67	0.39	0.7
	4-1	0.33	0.67	0.35	0.66	0.35	0.68	0.4	0.72
	1-4	0.23	0.64	0.24	0.63	0.22	0.61	0.24	0.67

BioInfer	CV	0.3	0.69	0.29	0.68	0.29	0.75	0.34	0.75
	4-1	0.3	0.69	0.32	0.68	0.25	0.65	0.31	0.7
	1-4	0.31	0.68	0.34	0.67	0.26	0.64	0.35	0.7

HPRD50	CV	0.58	0.69	0.59	0.73	0.44	0.72	0.56	0.73
	4-1	0.48	0.73	0.48	0.72	0.47	0.75	0.56	0.75
	1-4	0.33	0.67	0.31	0.64	0.26	0.63	0.3	0.65

LLL	CV	0.74	0.81	0.74	0.76	0.67	0.67	0.76	0.73
	4-1	0.43	0.68	0.44	0.71	0.31	0.6	0.39	0.74
	1-4	0.37	0.67	0.34	0.63	0.3	0.55	0.33	0.62

IEPA	CV	0.76	0.81	0.71	0.8	0.66	0.7	0.72	0.8
	4-1	0.35	0.69	0.32	0.64	0.19	0.56	0.29	0.68
	1-4	0.36	0.66	0.33	0.63	0.3	0.55	0.33	0.62

Precision plays a particularly important role in the interaction extraction task, because if the extracted information is processed by a biologist, she would not like the system if it produces too much rubbish. Therefore, we are particularly interested in the left side of the recall-precision chart, where precision is typically high, although recall may be quite low.

Table 4 shows the F-measure results calculated for classifier confidence threshold 0.5, as well as the AUC values which are not dependent on any threshold. Let us note that the F-measure values could be tuned up, because on most recall-precision charts precision does not drop significantly after a certain point, while recall keeps growing. However, this is not the aim of the current research, thus we leave the task of looking for the optimal operation point aside. Table 5 sums up our empirical observations over recall-precision curves provided in Figures 4, 5, 6, particularly on the area before 0.2-0.3 recall values. For example, Figure 4d clearly shows that lexical kernel performs best for the recall up to approximately 0.18 in this experimental setting. Using these empirical observations we fill Table 5. The table gives some insight on how different types of information affect the performance of the relation extraction system. Note that these observations are different from Table 4, because typically a 0.5 threshold covers more than 30% of recall. Below we provide a more detailed analysis of the results shown in Table 4 and in Figures 4, 5, 6.

**Table 5:** Information types

Data set	CV	4-1	1-4	Most used information
BioInfer	Lexical	Lexical	Lexical+Shallow	Lexical

LLL	Syntactic	Lexical+Shallow	Syntactic	Syntactic

HPRD50	Lexical	Lexical	Lexical	Lexical
		Lexical+Shallow		

IEPA	Lexical+Shallow	Syntactic	Syntactic	Biased to syntactic
		Lexical+Shallow		

AIMed	Syntactic	Lexical	Lexical	Lexical
		Lexical+Shallow		

Result	Not clear	Lexical+Shallow	Not clear	

The cross-validation setup reveals no clear leader for all data sets. For the LLL data set, the syntactic kernel shows the best performance (Figure 4a). That can be explained by the fact that the LLL data set is very small and contains relatively short hand-picked sentences with a simple syntactic structure. However, experiments with other data sets show that the lexical kernel gives the best results for the HPRD50 and BioInfer data sets (Figures 5b and 4b). In the case of BioInfer, this can be explained by the fact that the training set size is large enough to discriminate useful lexical features. For IEPA, the full kernel, i.e. lexical+shallow, performs best, while the lexical kernel shows the worst result (Figure 5a), and for the AIMed data set the syntactic kernel shows better results for small recall values (Figure 6a). The predictive power of deep syntactic features by themselves is very interesting, given that the lexical and lexical+shallow methods in theory can take additional advantage of the lexicon similarity within the same data set that is caused by the nature of the cross-validation set up.

When we train on 4 data sets and test on 1, the lexical+shallow kernel is among the best for all but the BioInfer data set. Figure 4d shows that the lexical kernel outperforms the others on BioInfer for small recall values. A significantly better performance of the lexical kernel for small recall values can be interpreted as a sign of overfitting, i.e. a classifier with a lexical kernel produces too specific patterns, which causes a successful classification of several instances, but is followed by a significant precision drop due to the unability to generalize over less frequent cases. On the other hand, other classifiers avoid overfitting and a steep precision drop, but at the cost of missing some very reliable patterns. Moreover, the lexical kernel shows a performance similar to the lexical+shallow kernel for HPRD50 and AIMed (Figures 5d and 6b), but fails on IEPA (Figure 5c). On the other hand, the syntactic kernel performs good on the IEPA and LLL data sets (Figures 5c and 4c), but is not that good on others.

Although with the 1-4 experimental setup there is no best kernel either, we can still observe some interesting patterns. The lexical kernel shows a significantly better performance on the AIMed and HPRD50 data set for small recall values (Figures 6c and 5f), while the syntactic kernel performs best for the LLL and IEPA data sets on the whole curve (Figures 4c and 5e). As it is shown on Figure 4e, training the classifier on LLL causes extreme curve shapes caused by the significant difference in size between the training set and the test data set. The first instances for the lexical+shallow and the shallow kernels were classified correctly, but further precision drops dramatically. After 0.25 recall value, the lexical kernel basically neglects all positive instances, and the curve shows simply the percentage of positive instances in the data set. Other kernels perform slightly better and the syntactic kernel is able to consistently outperform others. This can be explained by the fact that 80 sentences (the size of the LLL data set) is definitely not enough to train a classifier. Moreover, it shows that in the case of training information shortage the syntactic kernel can offer a better solution than others.

The last two experiments illustrate the case when the vocabulary of train and test data sets differ, which is often the case in the real world. In the former case the training set is large enough to successfully train the lexical+shallow kernel, making the difference in the vocabularies not so crucial. However, in the latter case, when the training set is much smaller than the test set (train on LLL case on Figure 4e) we can clearly see the influence of this fact on the performance difference between syntactic and lexical methods.

From the experiments above we can observe the following trends:

• lexical and combined methods are able to build better generalizations (due to large amount of available lexical data) and thus perform better with large (relative to test) training sets

• syntactic methods are able to achieve better results than lexical ones when the training set is small in comparison with the test set

Moreover, there seems to be a correlation between better performing kernels and data sets. For example, the syntactic kernel always obtains good results on the LLL and IEPA data sets, while the lexical+shallow kernel performs well for the BioInfer data set. Moreover, the lexical kernel is always on top for the HPRD50 data set. These observations show that the data set origin and properties (such as annotation strategy, average sentence complexity) have a strong influence on classifier performance.

Compiling ROC curves for one method on one chart allows us to analyze the robustness of this method on different data sets. In Figure 7 each chart displays ROC curves for one method for all experimental setups. The less spread the curves are in the ROC space, the more predictable the performance of the method is.

In most cases, the LLL cross-validation setup is out of the trend, because of its small size and density. Otherwise, the shallow (Figure 7c) and syntactic (Figure 7a) kernels exhibit more or less coherent behavior for all setups for the given data sets. The lexical+shallow kernel (Figure 7d) shows some spread, but again mostly due to the LLL data set's based setups, and the lexical kernel (Figure 7b) proves to be the most unpredictable.

## Conclusion

In this paper we examined different structured kernels with SVM's to study the impact of different features on the relation extraction process. We took four kernels that reflect different degrees of using syntactic and lexical information and performed three types of experiments to study the behaviour of these methods under different conditions. We performed our experiments on five benchmark data sets, being AIMed, BioInfer, IEPA, HPRD50 and LLL.

The most important observation is that by using only grammatical relations (syntactic kernel) we can obtain a similar performance as with an extended feature set (lexical kernel). This indicates the relative importance of grammatical information for the interaction extraction task. Another finding is the correlation between training/test set sizes and the method choice. We observed that when the training set is much smaller than the test set, then the syntactic kernel performs better. This might be explained by the fact that there are too few instances to induce useful lexical features, whereas syntactic features require less instances to produce better results.

When the training set grows, the performance of the full kernel becomes better, and when the training data set is larger than the test set (which rarely happens in real life), the full kernel outperforms all other kernels. From the stability point of view (i.e., the expected performance on unseen data), we can conclude that the syntactic kernel provides the best results, whereas the lexical kernel provides the worst results. The question of how different features within one feature type affect the quality of classification still remains open and represents an interesting direction for future work.

We believe that these findings can be helpful in building faster and less complicated classifiers, as well as for choosing a proper kernel according to the data set at hand.

## List of abbreviations

Abbreviations occured in figures: nsubj: nominal subject; dobj: direct object; nn: noun phrase; prep_*: preposition (with a corresponding name); det: determiner; amod: adjectival modifier; VBZ: verb, 3rd person singular present; NN: noun, singular or mass; NE1: 1st protein name; NE2: 2nd protein name.

## Authors' contributions

The results reported on in this paper are part of the PhD research of the main author, TF. MDC, CC and VH contributed as his PhD supervisors. All authors read and approved the document.
